# Uncovering the Role of Autochthonous Deteriogenic Biofilm Community: Rožanec Mithraeum Monument (Slovenia)

**DOI:** 10.1007/s00248-024-02404-0

**Published:** 2024-06-28

**Authors:** M. Ljaljević Grbić, Ivica Dimkić, Tamara Janakiev, Janez Kosel, Črtomir Tavzes, Slađana Popović, Aleksandar Knežević, Lea Legan, Klara Retko, Polonca Ropret, Nikola Unković

**Affiliations:** 1 University of Belgrade–Faculty of Biology, Studentski Trg 16, 11 000 Belgrade, Serbia; 2 The Institute for the Protection of Cultural Heritage of Slovenia, Poljanska Cesta 40, 1000 Ljubljana, Slovenia

**Keywords:** Decay, Endoliths, Microbes, Roman masterpiece, Stone relief, Subaerial biofilm

## Abstract

The primary purpose of the study, as part of the planned conservation work, was to uncover all aspects of autochthonous biofilm pertaining to the formation of numerous deterioration symptoms occurring on the limestone Rožanec Mithraeum monument in Slovenia. Using state-of-the-art sequencing technologies combining mycobiome data with observations made via numerous light and spectroscopic (FTIR and Raman) microscopy analyses pointed out to epilithic lichen *Gyalecta jenensis* and its photobiont, carotenoid-rich *Trentepohlia aurea*, as the origin of salmon-hued pigmented alterations of limestone surface. Furthermore, the development of the main deterioration symptom on the monument, i.e., biopitting, was instigated by the formation of typical endolithic thalli and ascomata of representative Verrucariaceae family (*Verrucaria* sp.) in conjunction with the oxalic acid-mediated dissolution of limestone. The domination of lichenized fungi, as the main deterioration agents, both on the relief and surrounding limestone, was additionally supported by the high relative abundance of *lichenized* and *symbiotroph* groups in FUNGuild analysis. Obtained results not only upgraded knowledge of this frequently occurring but often overlooked group of extremophilic stone heritage deteriogens but also provided a necessary groundwork for the development of efficient biocontrol formulation applicable in situ for the preservation of similarly affected limestone monuments.

**Supplementary Information:**

The online version contains supplementary material available at 10.1007/s00248-024-02404-0.

## Introduction

The irreplaceable legacy of material cultural heritage, associated with many values and meanings, such as aesthetic, artistic, historical, ethnological, scientific, or social, helps individuals, groups, and communities understand their surroundings, develop a sense of identity, and give meaning to a particular way of life [1–3]. An extensive list of raw materials is known to have been used throughout history to construct cultural, historical, and artistic objects and buildings, with stone being prevalent. Carbonate rock, such as limestone, was used for the earliest testimonies of human artistic expression and is one of the most commonly used materials globally since it is a soft, bright, and easy-to-carve rock [4]. However, limestone is continuously exposed to unstoppable degradation, with an estimated loss of approximately 1.5 to 3 mm of stone every 100 years in temperate climates, leading to permanent loss of priceless works of art around the world [5, 6]. In addition to this intrinsic decay, environmental factors and the “biosphere” further interact with constitutive materials altering their composition and structural properties, which means more severe damage is expected and the aesthetically unacceptable appearance of cultural artifacts is more expeditious [7]. Biological weathering of stone can be induced by all organisms living on/in it, from microorganisms to higher plants and animals, with special emphasis on well-known frequent limestone colonizers and exceptionally aggressive biodeteriogens: bacteria, algae, and fungi [6, 8]. They can develop independently, sometimes even in the form of mono-infestation, or more often unite and form multicolored subaerial biofilms, i.e., a single- to multilayer microbial assemblage of varying thickness at the interface between the atmosphere and rock in which the microorganisms are embedded in an extracellular matrix—EPS [9]. The growth of microorganisms and the development of biofilm alter the surface of the stone cultural heritage via several mechanisms, including (1) aesthetic disfigurement (e.g., pigment secretion), (2) physical disturbance (e.g., production of EPS that mechanically stresses the mineral structure, hyphal penetration, and growth of cells and trichomes that eventually increase the porosity of the rock), and (3) chemical reactions with materials (e.g., microbial excretion of inorganic/organic acids, and other “harmful” metabolites). Limestone, as a particularly bioreceptive substrate, is likewise gradually eroded by microbially induced solubilization and mineralization of calcium ions from minerals [9–11].

Understanding the complex microbial ecosystem of limestone cultural heritage is a prerequisite for the efficient control of microbial infestation responsible for documented biological damage so proper care and preservation for future generations can take place [12]. Various methods, both culture-dependent and culture-independent, are nowadays available for thoroughly characterizing these lithic communities, with a unanimous agreement among researchers that combining different approaches leads to a much deeper understanding. With pros and cons associated with each method, their combination allows their results to complement each other and generates more detailed information when profiling these communities, providing invaluable insights into the detection of previously unidentified biodeterioration agents [9, 13]. Obtaining information as detailed as possible on the footprint of thriving microbial communities is crucial for the selection of the most suitable conservation methods and tools to eliminate biodeteriogens without exacerbating biodeterioration and subsequently leading to the destruction of the material [13], or worse, directly damaging the stone matrices with unsuitable mechanical and/or chemical interventions.

With all that in mind, as part of the joined collaboration between the research groups from the University of Belgrade–Faculty of Biology and The Institute for the Protection of Cultural Heritage of Slovenia on a project “Novel biocides for cultural heritage of Southeast Europe–biocontrol and biomimetic systems for preservation of old masterpieces,” microbiome of a unique limestone relief, Rožanec Mithraeum monument in Judvoje forest in Slovenia, was studied using multitude of microscopic methods (widefield fluorescence microscopy, laser scanning confocal fluorescence microscopy, surface topography, FTIR and Raman spectroscopies, and optical and SEM microscopies) in combination with metabarcoding analysis, to gain improved comprehension of the complex microbial community and associated deterioration phenomena. In this way, a groundwork was established as the necessary first step for further research on the development of efficient biocontrol formulation applicable in situ for sustainable and long-term suppression of microbial infestation of this precious Roman masterpiece.

Two main hypotheses were formulated on the basis of observed distinctive deterioration symptoms on the limestone Rožanec Mithraeum monument (Slovenia): (1) salmon-hued pigmented alterations of limestone surface were the result of growth and metabolism of carotenoid-rich organisms and (2) origin of black dots observed within the pits is connected to microscopic lichens and/or rock-inhabiting microcolonial fungi.

## Study Site and Sampling Points

The Rožanec Mithraeum monument is located 4 km northwest of Črnomelj in the Judvoje forest above the village of Rožanec, Slovenia (45°36′26″N 15°09′53″E). Erected in the second century A.D. in a limestone wall of an old quarry in honor of the god Mithras, as part of the ancient religion of Mithraism, the monument is a 1.5 m tall carved relief of Mithra (Supplementary Fig. S1a) sacrificing the sacred bull (Supplementary Fig. S1b), watched by the sun (Supplementary Fig. S1c) and moon (Supplementary Fig. S1d) with a dog (Supplementary Fig. S1e), serpent (Supplementary Fig. S1f), and scorpion (Supplementary Fig. S1g) at his feet, the last two representing the forces of evil. The scene of sacrifice is accompanied by the priests Cautopates (Supplementary Fig. S1h) and Cautes (Supplementary Fig. S1i). Above the relief it is written in stone “*To the invincible god Mithras, Publius Elias: Nepotus, Proculus and Firminus, for your health and the health of your loved ones*.” Because it is placed within a forest, moisture content is high, and in multiple areas, there is a seepage of water from cracks within the rock. This promotes biological growth even on the macroscopic level, i.e., mosses and lichens. Recently, the monument has seen rapid decay due to the activity of a multitude of deteriogenic factors, with the clear evidence of deterioration obtained by comparison of its current state with a copy of the monument made in 1953 and now kept in the Bela Krajina Museum Metlika (BMM; belokranjski-muzej.si). Due to this, in 1956, a conceptual project for arranging this area was conducted by architect Gizela Šuklja (Jože Plečnik’s student), and in 2002, the Preliminary Conservation Program for the Restoration of the Monument was written [14]. In 2006, under the relief, excavation for drainage was completed, which prevented the rise of capillary water, and in 2007, a transparent canopy was installed above the relief, which reduces rain waterfall, limestone loading, and dirt accumulation. In 2008, a 3D scan of both the relief in nature and its replica in BMM was carried out to precisely evaluate how fast this monument is deteriorating [15–17]. Several extensive relief cleaning attempts were made, the last one being in 2011.

**Fig. 1:**
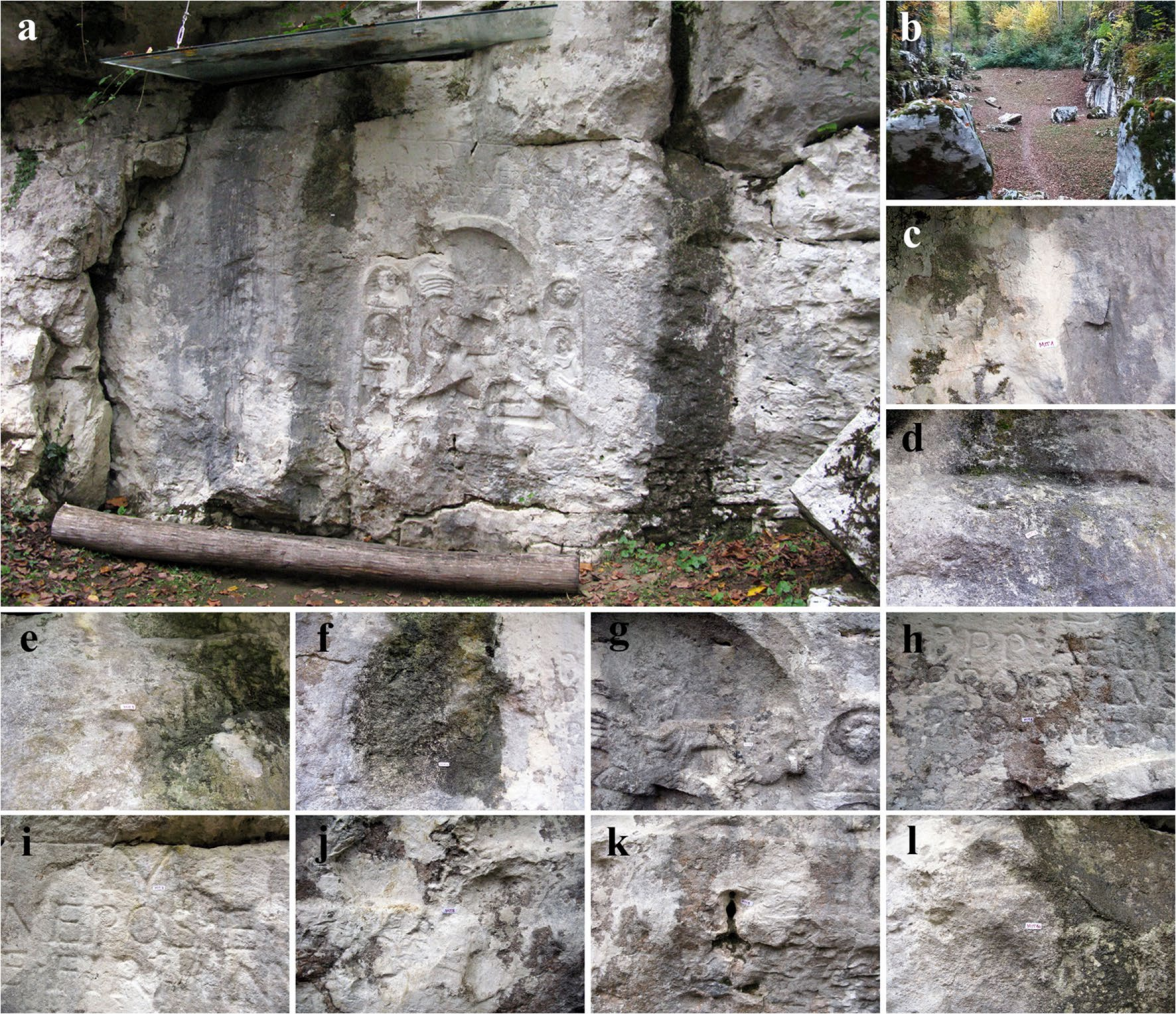
The Rožanec Mithraeum monument: **a** close-up view of relief carved in limestone wall; **b** immediate surroundings of the old quarry; sampling points characterized by pronounced symptoms of deterioration: **c** MIT1, **d** MIT2, **e** MIT3, **f** MIT4, **g** MIT5, **h** MIT6, **i** MIT7, **j** MIT8, **k** MIT9, and **l** MIT10

Sampling for the experiments in this study was carried out on 10 sampling points, i.e., five samples were taken from the relief and five from the surrounding limestone wall, all characterized by the presence of various alterations of the stone surface (Fig. 1 and Supplementary Fig. S2).

## Methods

### Environmental Parameter Survey

#### Moisture Content and Microclimate Measurements

Monument material moisture content (%), as well as air temperature (*T*, °C) and relative humidity (RH, %) of immediate surroundings, was measured at each sampling point using a testo 606–2 measuring instrument set to limestone mode.

### Characterization of the Deterioration Symptoms

For the characterization of the deterioration symptoms, microinvasive sampling was conducted. In order not to damage the relief itself, microinvasive sampling was conducted at the sampling point MIT1, which was outside the area of the sculpted stone. Moreover, stereomicroscopy showed that a large section of the stone monument was affected by biopitting and by red pigmented areas, which were also prevalent at sampling point MIT1; hence, there was no need to additionally damage the monument at other sampling points.

#### Preparation of Raw Microsamples

Raw microsamples (approx. 2 × 2 mm) were removed from the MIT1 sampling point of the Rožanec Mithraeum monument, using a scalpel and a hammer. For their cross-sectional analysis (needed only for widefield/confocal fluorescence microscopy analysis), the raw microsamples were embedded in polyester acrylic resin (Kristal PS, 2% (w/w), overnight solidification at room temperature, 1 h at 50 °C) and polished (RotoPol-15, Micromesh abrasive MD-Dac pads 320, 600, 800, 1200, 2400, and 4000 mesh).

#### Widefield Fluorescence Microscopy

Cross-sections of raw microsamples were stained using a Calcofluor White fluorescent dye (1:1 ratio with 10% KOH, 10 µL directly onto the cross-sectional surface, and 15 min of incubation at room T; two washing steps with distilled water), which specifically binds to the chitin cell wall and is used for highlighting fungal biofilms. Prior to and after the fluorescent staining, the sample cross-sections were photographed in the widefield fluorescence observation mode (Axio Imager.Z2m LSM 800, Zen Blue 2.5). The HXP unit (metal halide fluorescence light lamp module; 120 V) coupled with the filter set 49 (excitation: 365 nm, Beamsplitter: FT 395, emission: 445/50 nm) was used, and the DAPI channel (463 nm) was selected. The intensity of the lamp module was set to 15.0% and the shift value (under exposure time) was set to 80%. The same values (texp in ms) were used for photographs (Axiocam 503 color camera) taken before and after the fluorescent staining of a particular sample. Images were captured using the Axiocam 503 color camera.

#### Laser Scanning Confocal Fluorescence Microscopy

After the fluorescent staining, the cross-sections of raw microsamples were laser scanned by using the laser-scanning confocal fluorescent observation mode, within the Zeiss microscope system. The LSM 800 laser module LM URGB (contains fiber-coupled, pigtailed, and collimated lasers) was turned on and the anti-vibration table Vision IsoStation was pressurized using compressed air. Within the Acquisition tab of the Zen Blue 2.5 software, under the “LSM” screen (within the smart setup *menu*), the DAPI channel (463 nm) was selected. The intensity of the 405-nm DAPI-excitation diode laser (5 mW, class 3B) was set to 2%. Emission was detected with the LSM 800 MAT Confocal MA-detection module (main beam splitter (MBS), a variable pinhole with automatic alignment, two variable secondary dichroics (VSD) at a 10° angle to the incident beam for the most effective excitation light suppression, an emission filter in front of each of the two multi-alkali (MA) PMT confocal channel detectors). The pinhole was set to 1 Airy unit (AU) with an opening diameter of 36 µm. The digital gain of the PMT detectors was set to 0.9 (digital offset of 0); however, the master gain settings (in V) varied from sample to sample. Finally, by defining the scanning borders (set first/last), a 3D model was computer-generated from a Z-stack of images (Zen Blue 2.5 software), within an interval of 100 µm (each image 2.6 µm apart).

#### Surface Topography Scans

The raw microsamples (collected using a scalpel and a hammer) were directly adhered onto a glass slide. The surface topography was laser scanned, by using the 405-nm laser scanning mode, within the Zeiss microscope system (Zen Blue 2.5 software). Therefore, the same physical units and microscope system were applied as in “Laser Scanning Confocal Fluorescence Microscopy.” The intensity of the 405-nm violet diode laser (5 mW, class 3B), used for scanning, was set to 10%. The pinhole was set to 1 AU with an opening diameter of 25 µm. Even though the master gain settings varied from sample to sample, its values were always close to around 250 ± 10 V. In order to engulf the entire surface topography, the scanning borders (set first/last) were set to acquire a 700-µm long interval of Z-stack images (each image 0.41 µm apart) and these were saved as a CZI file format. This format was opened within the ImageJ distribution Fiji software, and the images were reduced to 8-bits. Finally, a 3D surface topography model was generated (3D Viewer option).

#### FTIR Spectroscopy

FTIR analysis of raw microsamples (collected using a scalpel and a hammer) was performed using FT-IR spectrometer TENSOR II and Hyperion 3000 infrared microscope equipped with Mercury Cadmium Telluride (MCT) detector cooled by liquid nitrogen. The specific microsamples were taken from the already removed samples and were placed between the windows of a diamond anvil cell and examined under FTIR microscope. Transmission FTIR spectra were acquired with 15 × objective, between 4000 and 600 cm^−1^ spectral range, with a resolution 4 cm^−1^ and 64 scans. Collected spectra were normalized and baseline corrected using OPUS software.

#### Raman Spectroscopy

Raman analysis of raw microsamples (collected using a scalpel and a hammer) was performed using a 532-nm laser excitation line with a Bruker’s SENTERA II dispersive Raman microspectrometer (Bruker Optics GmbH, Germany). Spectra were recorded using a 10 × objective and a 400 groove/mm grating, giving a spectral resolution of approximately 4 cm^−1^. A multi-channel, TE (thermo-electrically) cooled CCD detector was used, a power of 2.5 or 6.25 mW (10% or 25%, output laser power), integration time of 5 or 20 s with 2 accumulations.

### Characterization of Deteriogenic Microbial Community

#### In Situ Microscopy

Microbial growth and structural microscopic impairments of limestone surfaces were observed in the field using a portable Dino-Lite Edge digital microscope AM7915MZTL. Image processing and measurements were achieved with DinoCapture 2.0 v1.5.39.A software.

#### Optical Microscopy

Samples for optical microscopy were collected at each sampling point via the non-aggressive adhesive tape method [18]. Staining was done with Lactophenol Cotton Blue for the analysis of fungal biofilm constituents. For observation of cyanobacteria and algae on adhesive strips, as well as temporary slides, biofilm was mixed with a drop of glycerol. Samples were analyzed with a Zeiss Axio Imager M1 microscope using AxioVision Release 4.6 software. Identification of cyanobacteria and algae, based on morphological and ecological properties, was done according to [19–24].

#### Scanning Electron Microscopy (SEM)

Fragments of analyzed limestone substrata were collected via adhesive carbon tape on aluminum cylinders. SEM images were obtained at the University of Belgrade–Faculty of Mining and Geology using a JEOL JSM–6610LV microscope. Gold coating (*d* = 15 nm, *ρ* = 19.2 g/cm^3^) of samples was done with a Leica EM SCD005 sputter coater. Secondary electron and backscattered electron images were obtained using a W-filament gun, at 20 kV acceleration voltage in high-vacuum mode (15–30 µPa in the sample chamber) and magnifications ranging from 150 to 30,000 × .

#### Amplicon Sequencing

##### DNA Extraction, Library Preparation, and NGS Sequencing

Special sterile swabs (Puritan™ HydraFlock™) were taken from all of the sampling points and protected by DNA/RNA shields (Zymo Research) during transport. The extraction of ultra-pure DNA was completed using the Zymo BIOMICS DNA Mini Kit (Zymo Research) for several swabs from each sampling point, following the manufacturer’s protocol. The DNA yield was measured using Qubit Fluorometric Quantitation (Qubit 4 Fluorometer). Library preparation, using Nextera XT Index Kit (FC-131–1096), and amplicon sequencing step was performed using a 2 × 300 bp paired-end run on a MiSeq Sequencer, according to manufacturer’s instructions (Illumina) in commercially available service (Novogene, UK). To target the *ITS* II region, the primers were as follows: forward primer ITS3-2024F (5′-GCATCGATGAAGAACGCAGC-3′) and reverse primer ITS4-2409R (5′-TCCTCCGCTTATTGATATGC-3′).

##### Sequence Data Process, Taxonomy Annotation, and Phylogeny Inference.

Quality-based filtering/trimming was performed using the DaDa2 R package [25]. Reads were quality trimmed using default options in filterAndTrim with the addition: forward and reverse reads were right trimmed after 223 nt; reads shorter than 100 nt were discarded; all sequences having more than 2 for forward and 2 for reverse strand expected errors (calculated as sum(10^(− *Q*/10))—where *Q* is the quality score), were discarded (argument:maxEE = c(2, 2)). Sequence de-noising was performed in selfConsist mode where the algorithm alternates between sample inference and error rate estimation until convergence. Sequence pairs were merged with a minimum overlap of 12 nt (default). Chimera removal was performed using default options in removeBimeraDenovo with minFoldParentOverAbundance = 8 since it was shown that the default parameter overly eagerly flags sequences as chimeras in cases where there is a lot of diversity.

ITS taxonomy assignment was performed using the modified UNITE general FASTA release for eukaryotes 2 (10.17616/R31NJNIG) dev version. After removing all sequences shorter than 300 nt, two variants of the database were created: one where only sequences with at least family level annotation were kept (family unite) and another where sequences with at least class level annotation were kept (class unite). Both variants of the database were used to perform tax assignment up to the genus level using IDTAXA [26] with default options and threshold set to 50. The annotations from both databases were combined so that amplicon sequence variant (ASV) which did not have an assigned class, phylum, or kingdom using family unite was assigned these tax categories (if present) based on class unite annotations. After taxonomy inference, all ASV which were not classified as phylum: “Ascomycota,” “Basidiomycota,” “Bryophyta,” “Chlorophyta,” or “Rozellomycota,” were removed from further analysis. ASV corresponding to unidentified phyla were kept and reads with an unidentified kingdom were removed.

Prior to phylogeny inference, alignment of sequences was performed using DECIPHER R package [27]. Alignment was carried out for 5 iterations and 5 refinements. Phylogeny inference was conducted using FastTree 2.1.10 [28] using the following options: -spr 4—4 rounds in subtree-prune-regraft (SPR) moves -mlacc 2—always optimize all 5 branches at each NNI in 2 rounds -slownni—turn off heuristics to avoid constant subtrees -gtr—generalized time-reversible model -gamma—after optimizing the tree under the CAT approximation, rescale the lengths to optimize the Gamma20 likelihood NNI—minimum-evolution nearest-neighbor interchanges. For a detailed explanation of these options, see http://www.microbesonline.org/fasttree/. All genera present in at least 0.5% in any of the samples are presented in the relative abundance heat map position on top of the obtained unrooted phylogenetic tree. Each entry does not represent a single ASV but an aggregated tax category based on UNITE genus level tax annotations—for each tax category, the overall most abundant sequence was chosen as the representative for the respective tax category and its position in the phylogenetic tree was shown.

##### Statistical Analysis

Sequence diversity within samples (alpha diversity) was estimated using the phyloseq R package [29] at the ASV, genus, family, and phylum levels. Two sample Wilcoxon tests at ASV, genus, and family aggregations were used to compare the samples obtained from relief (MIT5–MIT9) and surrounding limestone wall (samples MIT1–MIT4 and MIT10). Alpha diversity was shown through estimators Shannon and Simpson. Observed and estimated richness was determined according to the following estimators: number of observations (OBS), Chao1, and ACE.

For visualization of beta diversity shared across sample communities at the genus level, a double principal coordinate analysis (DPCoA) [30] was used. Before analysis, all taxa with a read sum over all samples of less than 30 were removed. To test if there is significant separation between groups, the PERMDISP2 [31] was used. This method analyzes multivariate homogeneity of group dispersions (variances) on DPCoA distance matrix with adjustment for small sample bias in beta diversity estimates and lingoes correction to non-diagonal dissimilarities of the DPCoA distance matrix. After this, permutational multivariate analysis of variance using distance matrices (ADONIS) [32] was performed to analyze if two or more groups have similar compositions.

Differential abundance estimation was performed for all taxon ranks up to the genus level. Prior to differential abundance estimation, all taxa (genera) with a read sum over all samples of less than 30 were removed. Differential abundance estimation was performed using the microbiomeMarker package [33] using the DESeq2 [34] method as implemented in microbiomeMarker with default parameters. For *p*-adjustment, Benjamini and Hochberg method was used [35] and values of *p* < 0.01 were considered to be statistically significant.

Further annotation of inferred taxonomies was performed using FUNGuild [36]. The 395 identified fungal taxons (out of the 436 total taxons) at the genus level aggregation were annotated using FUNGuildR package (https://github.com/brendanf/FUNGuildR) to obtain ecological guild association.

All data were deposited within the NCBI database as BioProject ID: PRJNA1064956 (under the accessions from SRX23261750 to SRX23261759).

## Results

### Material Surface Moisture Content and Microclimate Parameters

Measured values of surface moisture content, temperature, and relative humidity are summarized in Supplementary Table S1. Surface moisture content values were in the range of 1.1 to 4.9%. The moisture content of limestone was for the most part uniform, ranging from 1.1 to 1.5%, with the exception of the two highest measured values of 4.9% (MIT4) and 2.5% (MIT10) that were documented on the parts of the monument where water drains from the upper edge of the stone wall. Surrounding air temperature and relative humidity were in the range of 13.8 to 14.9 °C and 60.3 to 65.7%, respectively.

### State of the Investigated Monument

The Rožanec Mithraeum monument was found in a dilapidated state, with apparent alterations of the limestone substrata induced over time by synergistic exertion of numerous deteriogenic factors. The main physical injuries to the relief include broken-off parts of the Mithra’s head, his arm holding a knife and his right extended leg; of the bull’s neck, of the dog’s and snake’s heads; and of the priest’s Cautopate’s left leg. Not all of the upper surroundings of the monument are stable and contain cracked pieces of rock that may fall and thus be potentially dangerous to the nearby visitors. These are marked by red circles in Supplementary Fig. S2. Relief surface, although for the most part still visible, is dominated by the abundant loss of limestone microfragments and is in various areas plagued by two distinct biodeterioration symptoms: diffused areas of salmon-hued pigmented alterations of the original stone coloration and microscale pits created in stone as a result of the bioerosion induced by different organisms, i.e., biopitting. Furthermore, the limestone wall that surrounds the relief on the left and right sides is quite moist due to leakage of water from the upper edge of the stone wall, which has thus created favorable conditions for the development of variously colored patinas and dense layers of highly developed subaerial biofilm in myriad shades of dark green and brown.

### Characterization of Dominant Deterioration Symptoms

**Fig. 2:**
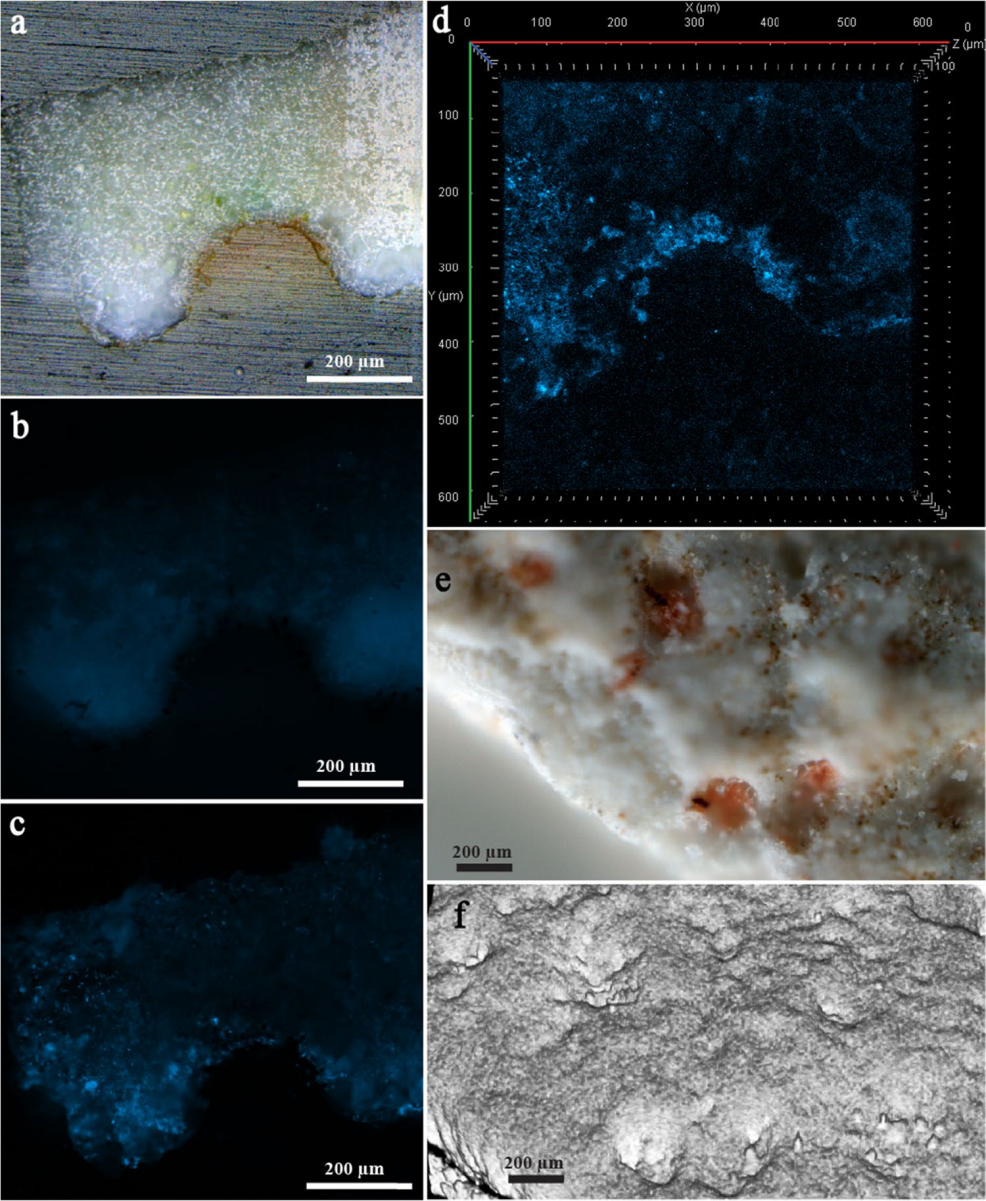
Raw microsample characterized by the presence of red pigment and isolated from MIT1: cross-section of this raw microsample was captured under the reflected-light brightfield observation mode (**a**); under the widefield fluorescence observation mode (prior to (**b**) and after (**c**) fluorescent staining); and under the laser scan with confocal fluorescence microscopy (**d**). 3D surface topography scanning was performed directly on the raw microsample’s surface (not on cross-section) (**e** and **f**)

Biodeteriorated raw microsample, collected from the salmon-hued pigmented areas of MIT1 and characterized by the presence of red pigment, is presented in Fig. 2a. After fluorescent staining of its cross-section, a slight fluorescence was observed on the edges of the pigmented area (Fig. 2c). Furthermore, fluorescence was also present on the upper layers of the polished stone’s surface (left and right of the pigmented area), which made it difficult to assess whether these parts were fungal in nature or were unspecific (pigment adhered to the porous surface of the stone material). However, under the more specific laser scanning, in confocal fluorescence observation mode, a bright and wide fluorescence was almost exclusively observed only around the edges of the pigmented area, i.e., the biofilm area, confirming that this alteration was of fungal origin (Fig. 2d). The 3D topography of the raw microsample’s surface revealed non-uniformly shaped micropits, from 70- to 250-µm deep (Fig. 2e, f).

**Fig. 3:**
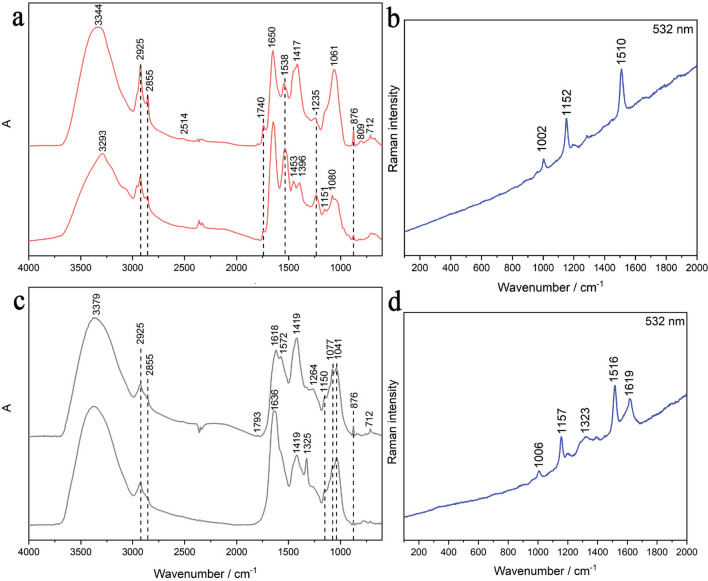
FTIR and Raman analyses of raw microsamples characterized by the presence of either red pigment (frames **a** and **b**) or biopitting phenomena (frames **c** and **d**). All raw microsamples were isolated from MIT1: **a** transmission FTIR spectra collected on “red dots”; **b** Raman spectrum collected on “red dots”; **c** transmission FTIR spectra collected on “black dots”; **d** Raman spectrum collected on “black dots”

Transmission FTIR spectra, collected on red pigmented areas of the raw microsample from MIT1 (Fig. 2e), are presented in Fig. 3a. Both FTIR spectra show characteristic structural protein amide bands placed at 3293 (amide A), 1650 (amide I), 1538 (amide II), and 1235 cm^−1^ (amide III). These peaks along with the strong signal between 1130 and 1000 cm^−1^ indicate the presence of chitin. Bands at 2925, 2855, and 1740 cm^−1^ suggest the presence of lipid components. In the FTIR spectra, calcium carbonate (calcite) (2514, 1793, 1417, 1396, 876, and 712 cm^−1^) bands were also detected. In addition, Raman analysis was also performed on the red pigmented areas of this raw microsample (from MIT1). The Raman spectrum (Fig. 3b) obtained from the circular formations in the areas seen as salmon-hued in optical microscopy (they were carefully removed for the transmission FTIR microscopic investigations) shows bands at 1510 (C = C stretching), 1152 (C–C stretching), and 1002 cm^−1^ (C = CH bending), which are characteristic carotenoid Raman skeletal features [37]. This result indicates the presence of a microbial infestation [38].

**Fig. 4:**
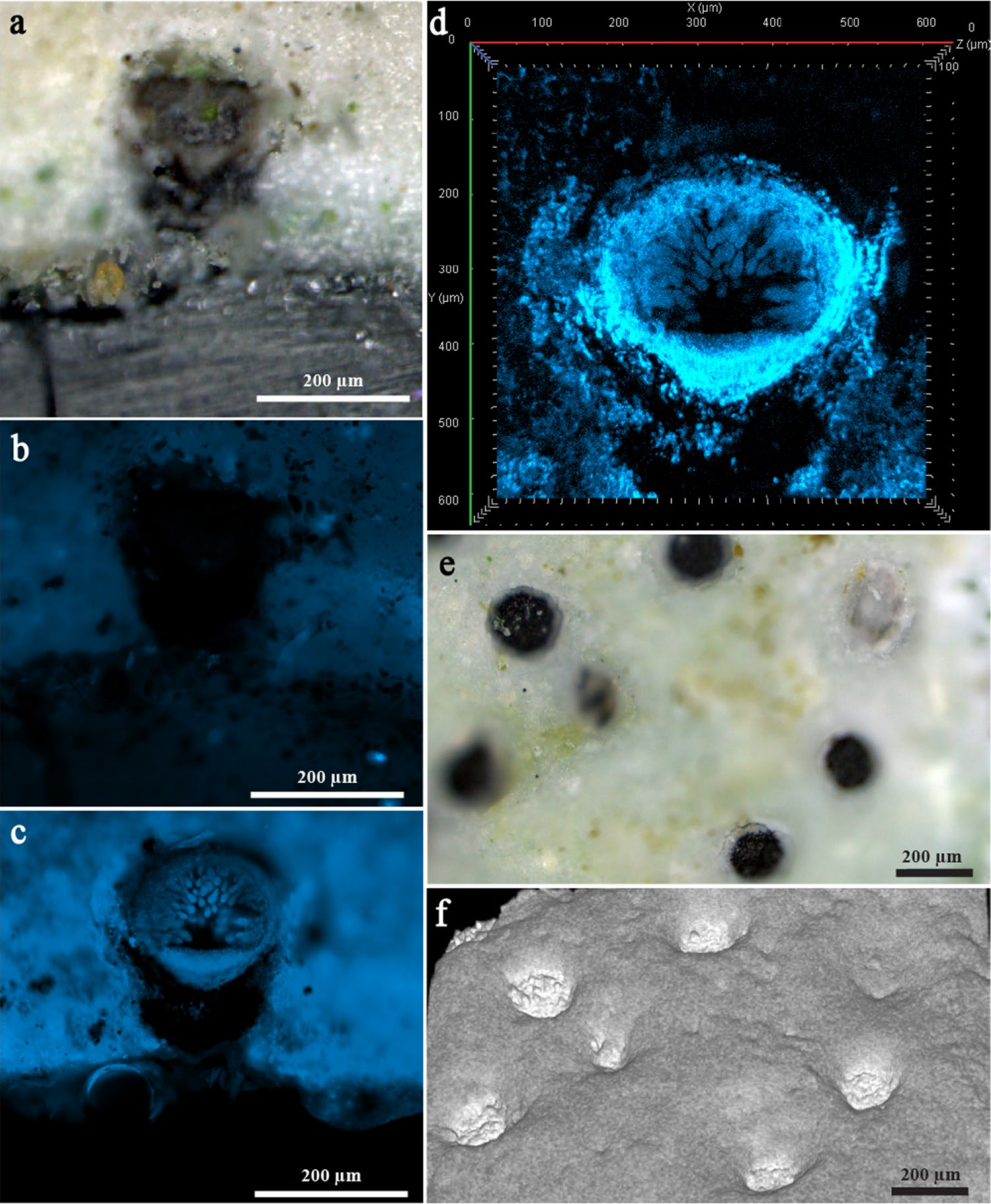
Raw microsample characterized by the presence of biopitting symptoms and isolated from MIT1: cross-section of this raw microsample was captured under the reflected-light brightfield observation mode (**a**); under the widefield fluorescence observation mode (prior to (**b**) and after (**c**) fluorescent staining); and under the laser scan with confocal fluorescence microscopy (**d**). 3D surface topography scanning was performed directly on the raw microsample’s surface (not on cross-section) (**e** and **f**)

Biodeteriorated raw microsample, collected from MIT1 and characterized by the presence of biopitting, is presented in Fig. 4a. After fluorescent staining of its cross-section, numerous chitin containing fungal structures, subglobose in shape, became visible within the center of the pits (Fig. 4c), which was prior to staining completely dark in the widefield fluorescence observation mode (Fig. 4b). Staining with Calcofluor White dye, which specifically binds to the chitin within the fungal cell wall, enabled the clear perception and the confirmation of the fungal origin of structures within the center of the pits, i.e., perithecia. Laser scanning confocal fluorescence observation mode further outlined the details: inward orientation (toward the center of the pit), well-developed peridium, and the hymenium layer with asci (Fig. 4d). The 3D topography of the raw microsample’s surface revealed strong and deep (~ 100 µm) micropits which were circular in shape (diameter of ~ 150 µm), with each of their bottom sides being filled with single perithecium (Fig. 4e, f).

FTIR spectra collected on black biopitting dots of the raw microsample from MIT1 (Fig. 4e) reveal distinctive absorption bands of oxalates placed at 1636 and 1325 cm^−1^ (Fig. 3c) [39]. Detection of oxalates, excreted by microorganisms [40], in the black dots suggests that oxalic acid is probably the most responsible for pitting on the surface of the calcium carbonate substrate. Namely, FTIR analysis showed that the substrate consists of calcium carbonate—calcite (IR bands at 1793, 1419, 876, and 712 cm^−1^). Signals in the region between 1200 and 1000 cm^−1^ indicate the presence of polysaccharides. An extra FTIR band placed at 1572 cm^−1^ suggests the presence of carboxylates. From the FTIR spectrum which contains a newly formed band at 1572 cm^−1^, it is clearly visible that the absorption band with a maximum at 1419 cm^−1^ becomes stronger. These changes are in agreement that free carboxylic acid groups of melanized fungal structures are converted to carboxylate under certain conditions [41, 42].

In addition, Raman analysis performed on the black biopitting dots of this raw microsample (from MIT1) (Fig. 3d) revealed the presence of carotenoids by the characteristic bands at 1516 (C = C stretching), 1157 (C–C stretching), and 1006 cm^−1^ (C = CH bending) [37]. An additional signal (namely, broader bands at 1619 and 1323 cm^−1^) was also observed, but its assignment is not entirely clear. It could indicate the presence of a carbonaceous substance and/or the presence of a melanin-like metabolite [43–45].

### In Situ Detection of Surface Microbial Growth and Stone Alterations

**Fig. 5:**
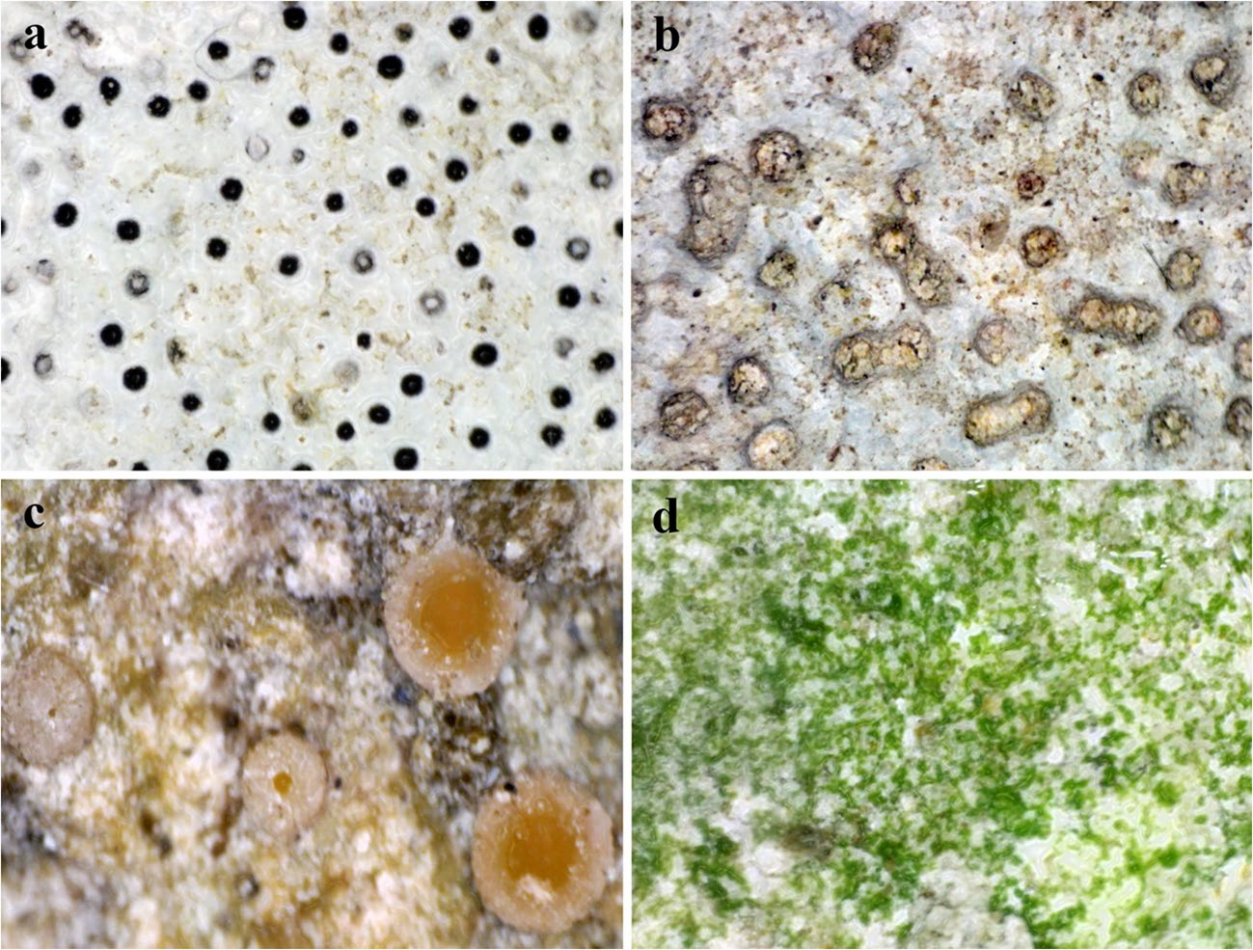
In situ microscopy of deteriorated surfaces of Rožanec Mithraeum monument: **a** microscale pits mostly filled with visible melanized perithecia of *Verrucaria* sp.; **b** biotroughs; **c** pale orange apothecia of *Gyalecta jenensis*; **d** diffuse red-pigmented alterations of limestone substrata; **e** green biofilm

Direct observation of the relief limestone surface, via portable in situ microscope, revealed the presence of visible microbial growth, in the form of scattered pale orange ascomata-apothecia belonging to crustose epilithic lichen *Gyalecta jenensis* (Fig. 5c) and diffuse green-hued biofilm dominated by phototrophic microorganisms (Fig. 5e). Furthermore, various alterations of the limestone surface were also observed with the predominant symptoms being diffuse salmon-hued pigmented areas (Fig. 5d) and symptoms of the biopitting phenomenon, i.e., bioerosion-induced microscale pits of various sizes, in the form of biotroughs—empty pits of various sizes (Fig. 5b) or with visible melanized ascomata-perithecia resembling typical pyrenocarpous representatives of *Verrucaria* genus (Fig. 5a).

### Thriving Epilithic Microbial Community

Microscopic analysis of the sampled biofilms revealed a predominance of phototrophic microorganisms belonging to Cyanobacteria, Chlorophyta, and Bacillariophyta (Supplementary Table S2, Supplementary Fig. S3). Cyanobacteria were the most diverse with 13 genera, with the most taxa found in *Gloeocapsa*, *Chroococcus*, and *Gloeothece*, followed by representatives of *Leptolyngbya*, *Aphanocapsa*, and *Aphanothece* genera. *Aphanocapsa muscicola* was the only identified taxon documented at nearly all sampling sites. The highest number of cyanobacterial taxa was found at MIT2, MIT4, and MIT10, i.e., parts of the monument where water drains from the upper edge of the stone wall, while the lowest number was documented at MIT7. Chlorophyta were less diverse than Cyanobacteria with species from 8 genera recorded, along with green algae in mass and lichen photobionts. *Desmococcus olivaceus* (Supplementary Fig. S3a) and *Trentepohlia* sp., in the form of lichen photobionts, were found at most sampling points. Of the Bacillariophyta, only two representatives, *Orthoseira roeseana* and *Pinnularia* sp., were observed in fresh biofilm material. On the other hand, heterotrophic biofilm constituents were, although present, much less represented, with occasional fungal propagules (Supplementary Fig. S3f) deposited either from the air or via drained water and numerous melanized mycobiont branched septate hyphae observed permeated through the limestone substrata (Supplementary Fig. S3e). Of notable importance is the presence of fragments of pigmented upper cortex of endolithic lichen (Supplementary Fig. S3d).

**Fig. 6:**
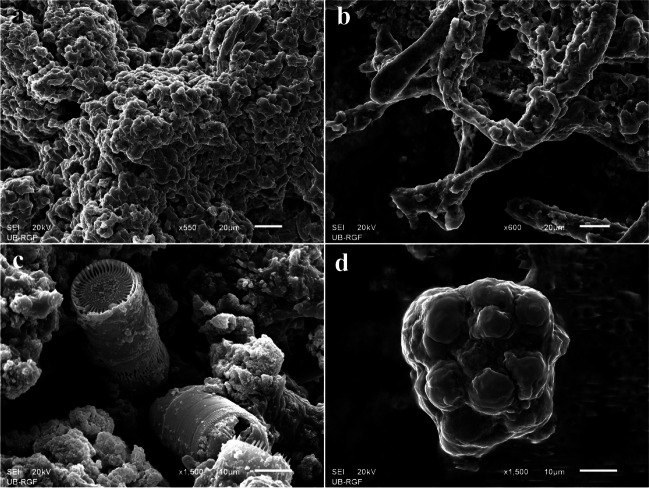
Scanning electron micrographs of fragments from deteriorated surfaces of Rožanec Mithraeum monument: **a**, **b** details of epilithic lichen thallus; **c** vegetative lichen propagule—soredium; **d** diatom *Orthoseira roeseana*

Observations made with optical and in situ microscopy were additionally confirmed with SEM: interwoven mass of epilithic lichen’s photobiont and mycobiont (Fig. 6a, b) and vegetative propagule (Fig. 6c), as well as details of *Orthoseira roeseana* permeating the limestone substrata (Fig. 6d).

### Total Fungal and Green Algae Community

**Fig. 7:**
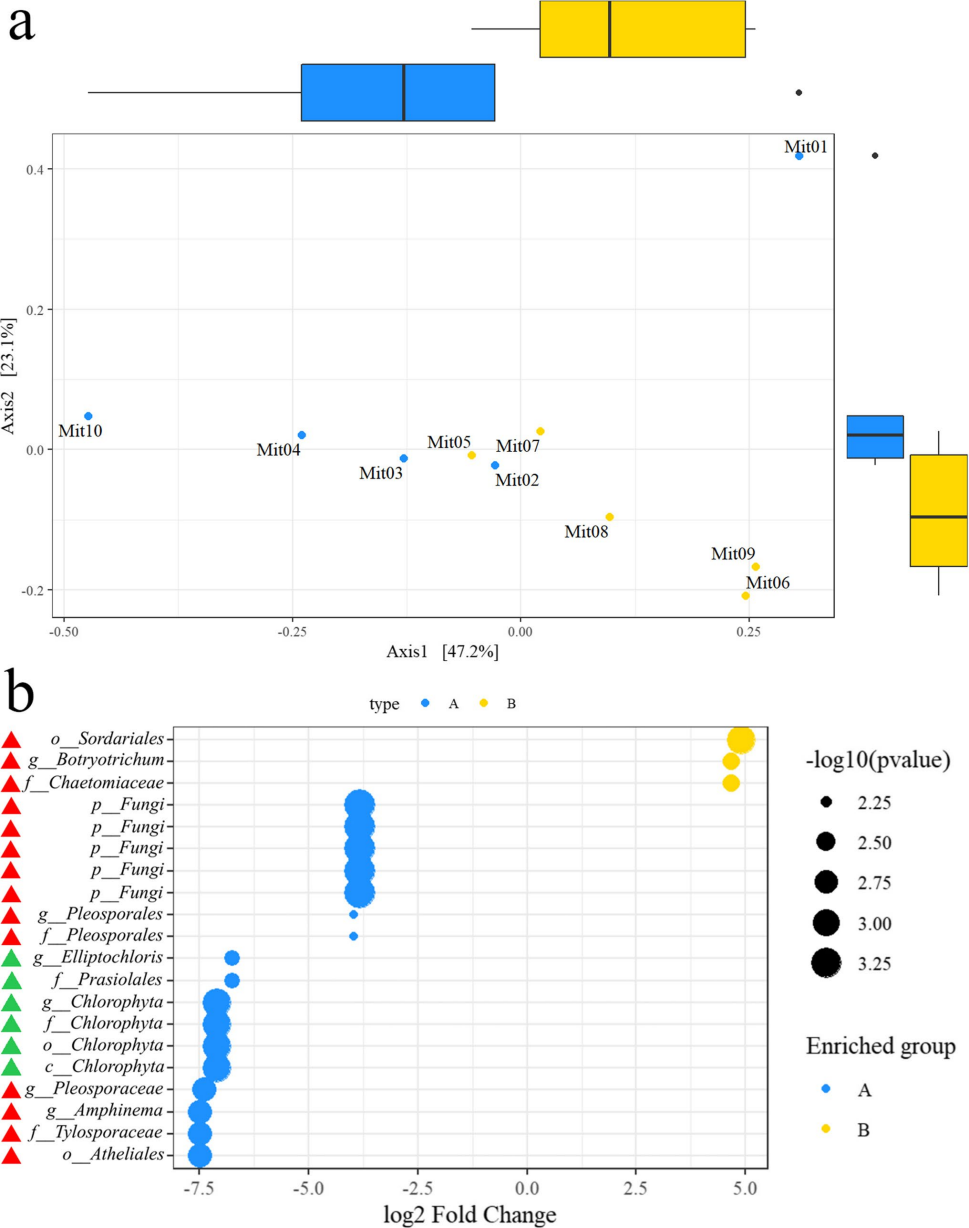
Beta diversity of fungi and green algae communities (**a**) and differential abundance of fungi—red triangles and green algae—green triangles (**b**) within samples from the relief (yellow) and surrounding limestone wall (blue) of the Rožanec Mithraeum monument. *p*-adjusted values < 0.01 according to Benjamini and Hochberg method were considered statistically significant

A total of 1,066,413 raw sequences were obtained from the ITS libraries sequencing (from 100,947 to 117,676 per sample). After denoising, quality filtering, and length trims, the number of reads ranged from 50,728 up to 82,042 (Supplementary Table S3). Based on the estimated alpha diversity, no statistically significant differences in fungal and green algae communities between the relief and surrounding limestone wall were observed (Supplementary Fig. S4). From the beta diversity, presented in Fig. 7a, these two communities, from the relief and surrounding limestone area, are clearly compositionally different (*p* value = 0.071) even though there are “out” groups, i.e., samples MIT1 and MIT5. Axis 1 indicates that there was a 47.2% variability among the studied samples.

**Fig. 8:**
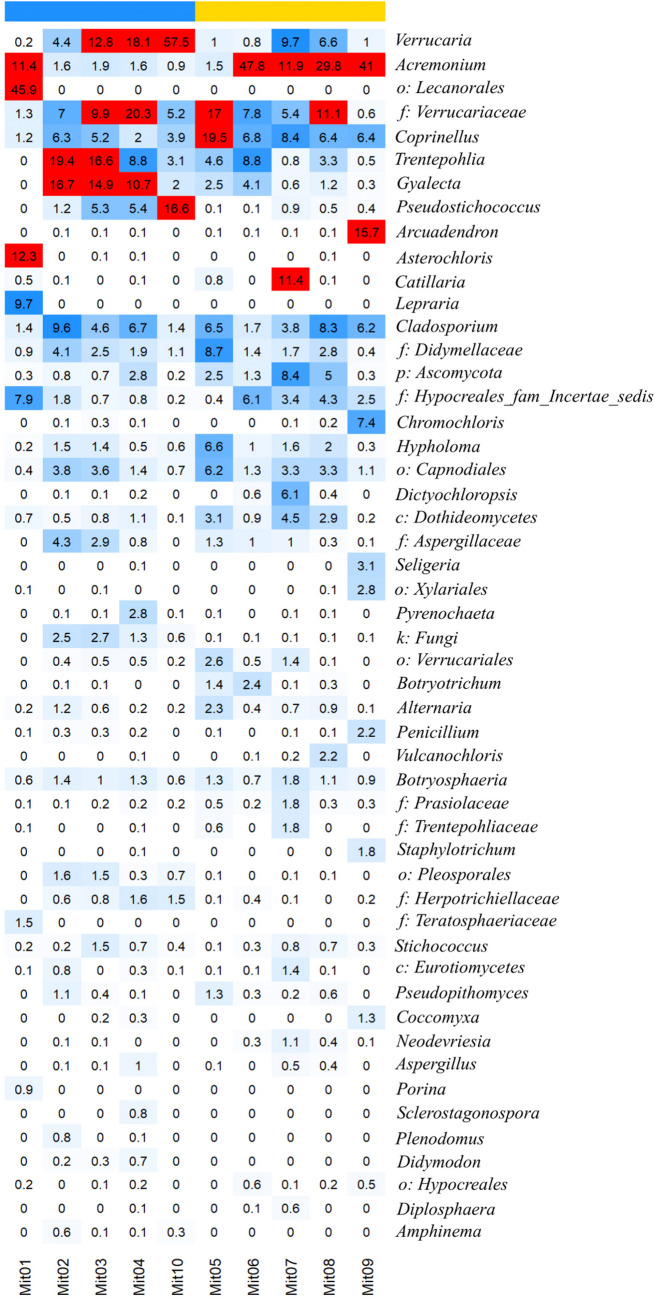
The relative abundance (%) of fungal and green algae taxa from the relief (MIT5–MIT9, top yellow bar) and surrounding limestone wall (MIT1–MIT4 and MIT10, top blue bar) of the Rožanec Mithraeum monument. The white-blue color gradient represents a linear gradient mapped to 0–10% relative abundance, while the red color indicates a relative abundance over 10%

The phylum Ascomycota predominated all samples (64.63–85.35%), followed by the phylum Basidiomycota (1.57–28.90%) (Supplementary Fig. S5). The phylum Chlorophyta was likewise present in a high percentage (6.08–24.62%) with algae of genus *Trentepohlia*, common lichen photobiont, dominant in almost all investigated samples (0.5–19.4%). The most abundant fungi in samples from the surrounding limestone wall (MIT1–MIT4 and MIT10) were *Verrucaria*, with a relative abundance of 18.6%, *Gyalecta* (8.9%), an unidentified genus from Verrucariaceae family (8.7%), *Cladosporium* (4.7%), *Coprinellus* (3.7%), and *Acremonium* (3.5%) (Fig. 8). All relative abundance values shown are average values of five repetitions. On the other hand, in samples taken from the relief (MIT5–MIT9), the highest relative abundance of fungi was documented for *Acremonium* (26.4%), *Coprinellus* (9.5%), an unidentified genus from Verrucariaceae family (8.4%), *Cladosporium* (5.3%), *Verrucaria* (3.8%), *Catillaria* (2.5%), and *Hypholoma* (2.3%). All relative abundance values shown are average values of five repetitions.

The most statistically significant, abundant, or unique, microbial taxa on the surrounding limestone wall were representatives of the genus *Amphinema*, the family Tylosporaceae, an unidentified family from the order Pleosporales, the orders Atheliales and Prasiolales, the phylum Chlorophyta, and unidentified representatives of the kingdom Fungi. On the other hand, on the relief, the most statistically significant, abundant or unique, were genus *Botryotrichum*, family Chaetomiaceae, and order Sordariales (Fig. 7b).

The relative abundance of various ecological groups of fungi is presented in Supplementary Fig. S6a. Most of the fungi on the surrounding limestone wall are *lichenized* fungi. Furthermore, to a considerable extent, *animal pathogen-endophyte-fungal parasite-plant pathogen-wood saprotroph* and *dung saprotroph-plant saprotroph-wood saprotroph* groups were also present. The most abundant ecological group on the relief was *animal pathogen-endophyte-fungal parasite-plant pathogen-wood saprotroph*, followed by *lichenized* fungi and *dung saprotroph-plant saprotroph-wood saprotroph*. On the other hand, based on the trophic mode, fungi from the *symbiotroph* group dominated the limestone wall around the relief (Supplementary Fig. S6b). In addition, to a smaller extent, *pathotroph-saprotroph-symbiotroph*, *saprotroph*, *pathotroph-saprotroph*, and *pathotroph-symbiotroph* groups were present. On the relief, *pathotroph-saprotroph-symbiotroph* group dominated, while to a lesser extent, an abundance of *saprotroph*, *symbiotroph*, and *pathotroph*-*saprotroph* was present as well.

## Discussion

The origin of (lime)stone biopitting was always a matter of debate among researchers due to the difficulty of determining whether the organisms documented inside the pits have actively contributed to pit formation or merely occupied more favorable ecological conditions [46]. Among the various organisms linked to the biopitting phenomenon, cyanobacteria are the most frequently reported, followed by fungi (including lichens), and finally green algae. Cyanobacteria are often the first colonizers of exposed limestone surfaces [5, 47] and are considered strong deteriogens that directly cause aesthetic, physical, and chemical impairments and indirectly promote the development of other microorganisms [6]. An abundance of cyanobacteria on limestone monuments is generally related to the favorable microclimatic parameters and the alkaline nature of the limestone that favors their growth [48]. They tend to dominate and form thick biofilms on moist surfaces and areas [5], consistent with presented results where the highest diversity of cyanobacterial taxa was documented at MIT4 and MIT10, i.e., in areas of the monument where water drains from the top of the stone wall. *Gloeocapsa*, *Chroococcus*, and *Gloeothece* were the most diverse cyanobacterial genera, all three of whom are mentioned in the literature as common inhabitants of limestone monuments [5], with *Gloeocapsa* considered the most frequently recorded genus [13, 49]. Some cyanobacterial taxa could not be determined but are of notice due to the presence of calcified sheaths. The latter is considered an important mechanism for the degradation of calcareous rocks due to the migration and precipitation of calcium on cyanobacterial cells [5]. With regard to carbonate rock, however, lichens are the most frequently cited colonizers and powerful deteriogens [46]. Lichenized fungi of the Verrucariaceae family have a pronounced tendency to colonize limestone and subsequently induce the formation of the pits, with ascomata of *Verrucaria baldensis*, *V. biatorinaria*, *V. buschirensis*, *V. fuscella*, *V. marmorea*, and *V. rubrocincta* previously documented on several limestone heritage sites [46, 50–53]. In the present study, lichenized fungi of the *Verrucaria* genus were among the most abundant organisms documented via metabarcoding analysis in the samples both from the surrounding limestone wall and the relief, with average relative abundances of 18.6% and 3.8%, respectively. Furthermore, other endolithic and epilithic lichens were also present in high percentage on the surrounding limestone wall (*Gyalecta*—8.9% and unidentified genus from Verrucariaceae family—8.7%) and the relief (an unidentified genus from Verrucariaceae family—8.4% and *Catillaria*—2.5%). The domination of lichenized fungi, both on the relief and surrounding limestone, is further supported by the high relative abundance of *lichenized* and *symbiotroph* groups in FUNGuild analysis, a software tool used to predict fungal functions in the environment [54]. However, fully developed ascomata of only two lichenized fungi were documented on/in limestone substrata via different microscopic analyses, i.e., apothecia of *Gyalecta jenensis* and perithecia of *Verrucaria* sp., marking them as the main fungal deteriogens of the investigated Roman monument. The diffuse presence of *G. jenensis* apothecia can point out the origin of salmon-hued pigmented alterations of limestone surface, where the presence of carotenoids was documented via FTIR and Raman analyses, as photobiont of this lichen is typically subaerial green algae *Trentepohlia aurea* that accumulates large quantities of various carotenoids in the filaments which protects them from ultraviolet light or high irradiance [55, 56]. This green alga was, based on metabarcoding analysis, present in almost all investigated samples with high relative abundance (0.5–19.4%). Based on the literature data, trentepohlialean algae can induce typical red-hued discolorations and micropits either as free-living, lichen-like colonies [57] or as photobiont in epilithic lichens [55]. Red pigmented micropits observed on the deteriorated surfaces of the limestone Rožanec Mithraeum monument are most probably caused by the production of carotenoids by *T. aurea* as a photobiont of *G. jenensis*. Alternatively, nitrogen-limited conditions could decrease chlorophyll and phycocyanin and increase carotenoid content in some cyanobacteria, especially in representatives of *Gloeocapsa* [49], which could contribute to their general recognition by FTIR and Raman analyses. Accumulation of carotenoids was already linked to the development of red hues on limestone heritage, i.e., sculptures from the Portuguese National Museum of Ancient Art and historic buildings at the Mayan site of Edzna, Mexico [38, 57, 58].

In addition to aesthetically impairing the monument, pigmented alteration may absorb more sunlight, which increases physical stress by expansion and contraction caused by temperature changes [5]. These microbially induced alterations of limestone’s original coloration make cleaning procedures challenging as pigmentation can be irreversible or cleaning can induce irreversible damage [59]. Furthermore, to the best of our knowledge, this is the first record of *G. janensis* on a limestone heritage site and its potential to induce mechanical weathering of stone monuments is still mostly unknown, as epilithic lichen can generally exert deleterious effects to various degrees: (1) no or negligible hyphal penetration, (2) penetration along pre-existing fractures and cleavage planes of minerals, or (3) major hyphal penetration within the rock matrix [51]. On the other hand, based on all of the presented results, the deteriogenic effect of *Verrucaria* sp. on the Rožanec Mithraeum monument is apparent, with numerous pits approximately 100 (*d*) × 200 (*w*) µm filled with melanized perithecia. In numerous areas, the death of *Verrucaria* sp*.* occurred, and fruiting bodies detached, leaving empty pits in limestone that progressively got enlarged by water (e.g., rainfall, water runoff, water accumulation) or further lithobiontic activity and coalesced forming larger interconnected depressions known as biotroughs (Fig. 7b) that caused the partial break or detachment of stone [60]. Furthermore, FTIR analysis pointed out the presence of oxalates in the pits suggesting that oxalic acid mediated in the dissolution of carbonate rock. According to Adamo and Violante [61] and Bungartz et al. [62], despite it being one of the most active agents of stone chemical alteration, its production in endolithic lichens, unlike epilithic species, does not seem to be a distinctive characteristic and was to date detected only in one closely related species, *V. rubrocincta*. Dissolution of carbonates by endolithic lichens is achieved via yet an unknown mechanism albeit several means were proposed: (1) secretion of chelating substances, (2) release of respiratory carbon dioxide, and (3) production of siderophore-like compounds [63]. Moreover, the acidic environment caused by these exudates further promotes limestone degradation. The biofilm observed not only supports the growth and function of the said taxa but also creates the microenvironment allowing for a sufficient concentration of the organic acids and the lowered pH localization with an extracellular matrix—EPS [9].

The Rožanec Mithraeum monument represents an emblematic case of a Roman heritage site with the main conservation issue being the result of colonization by endolithic and epilithic lichens. Even though a thriving microbial community, predominated by phototrophic microorganisms, is indeed present on the investigated monument, distinctive deterioration symptoms observed via different methods are in fact associated with fully developed thalli of two lichenized fungi: epilithic *Gyalecta jenensis* and endolithic *Verrucaria* sp. Furthermore, in spite of being one of the most frequent colonizers of limestone monuments, endolithic lichens have been much less studied in depth compared to epilithic, for the most part, due to their inconspicuous growth and color, which as a result has them overlooked and not recognized by specialist involved in stone conservation. This has de facto resulted in a gap of knowledge regarding the cleaning method and biocides potentially applicable in limestone conservation for the efficient removal of these extremophilic organisms. To select an effective procedure, in-depth knowledge is needed on thriving stone biodeteriogens and any chemical and mechanical alterations induced by their presence and activity. As such, determining the main agents of the decay of the investigated Roman masterpiece provided a necessary groundwork for the development of efficient biocontrol formulation, based on the metabolites of GRAS group beneficial bacteria, applicable in situ for sustainable and long-term suppression of epilithic and endolithic lichens, with the ultimate goal to extend knowledge and design suitable biocontrol/conservation strategies to preserve similarly affected limestone monuments.

### Supplementary Information

Below is the link to the electronic supplementary material.

Supplementary file1 (RAR 15898 KB)

## Data Availability

No datasets were generated or analyzed during the current study.

## References

[1] UNESCO 2007. Safeguarding intangible heritage and sustainable cultural tourism: opportunities and challenges. UNESCO-EIIHCAP Regional Meeting. https://unesdoc.unesco.org/ark:/48223/pf0000178732 Accessed 15 January 2024

[2] UNESCO 2009. UNESCO framework for cultural statistics. Institute for Statistics. https://uis.unesco.org/sites/default/files/documents/unesco-framework-for-cultural-statistics-2009-en_0.pdf Accessed 15 January 2024

[3] Mekonnen H, Bires Z, Berhanu K (2022). Practices and challenges of cultural heritage conservation in historical and religious heritage sites: evidence from North Shoa Zone, Amhara Region. Ethiopia Herit Sci.

[4] Pinheiro AC, Mesquita N, Trovão J, Soares F, Tiago I, Coelho C, de Carvalho HP, Gil F, Catarino L, Piñar G, Portugal A (2019). Limestone biodeterioration: a review on the Portuguese cultural heritage scenario. J Cult Herit.

[5] Scheerer S, Ortega-MoralesO GC (2009). Microbial deterioration of stone monuments—an updated overview. Adv Appl Microbiol.

[6] Polo A, Gulotta D, Santo S, Di Benedetto C, Fascio U, Toniolo L, Villa F, Cappitelli F (2012). Importance of subaerial biofilms and airborne microflora in the deterioration of stonework: a molecular study. Biofouling.

[7] Tiano P (2002) Biodegradation of cultural heritage: decay mechanisms and control methods. Proceedings of the ARIADNE Workshop 9 - Historic materials and their diagnostic, Prague

[8] Gorbushina AA (2007). Life on the rocks. Environ Microbiol.

[9] Cappitelli F, Villa F, Polo A (2014) Culture-independent methods to study subaerial biofilm growing on biodeteriorated surfaces of stone cultural heritage and frescoes. In: Donelli G (ed) Microbial biofilms: methods and protocols, Vol. 1147 Springer Science+Business Media, New York, pp 341–366. 10.1007/978-1-4939-0467-9_24 10.1007/978-1-4939-0467-9_24 24664845

[10] Krumbein WE (1992) Colour change of building stone and their direct and indirect biological causes. In: Delgado Rodriguez J, Henriques F, Telmo Jeremias F (eds) Proceedings of 7^th^ International Congress on Detection and Conservation of Stone, LNEC, Portugal, pp 443–452.

[11] Hsieh P, Pedersen JZ, Bruno L (2014). Photoinhibition of cyanobacteria and its application in cultural heritage conservation. Photochem Photobiol.

[12] Warscheid T, Braams J (2000). Biodeterioration of stone: a review. Int Biodeter Biodegr.

[13] Gutarowska B, Celikkol-Aydin S, Bonifay V, Otlewska A, Aydin E, Oldham AL, Brauer JI, Duncan KE, Adamiak J, Sunner JA, Beech IB (2015). Metabolomic and high-throughput sequencing analysis—modern approach for the assessment of biodeterioration of materials from historic buildings. Front Microbiol.

[14] Breščak D (2002) Mitrej pri Rožancu. Rast 13. The Institute for the Protection of Cultural Heritage of Slovenia, Ljubljana

[15] Jež J, Milanič B (2018) Geological survey of the problem of dissolution of the relief of the ancient Mithraeum above Rožanec. GeoZS Geological Survey of Slovenia, Ljubljana

[16] Lazzarini L (2021). Evaluation of the condition and proposals for the preservation of the monument: the conservation of the stone of the Mithraeum‘s relief of Rožanec.

[17] Lazzarini L, Žbona N (2021) Macroscopic examination of the condition and damage mapping according to the recommendations in NorMaL – 1/88. Iuav University of Venice and The Institute for the Protection of Cultural Heritage of Slovenia, Ljubljana

[18] Urzì C, de Leo F (2001). Sampling with adhesive tape strips: an easy and rapid method to monitor microbial colonization on monument surfaces. J Microbiol Methods.

[19] Komárek J (2013) Cyanoprokaryota. 3. Teil: Heterocytous Genera. In: Büdel B, Gärtner G, Krienitz L, Schagerl M (eds) Süβwasserfora von Mitteleuropa, Vol. 19/3 Springer Spektrum, Berlin, pp 1–1130

[20] Komárek J, Anagnostidis K (1998) Cyanoprokariota. 1. Teil: Chroococcales. In: Ettl H, Gärtner G, Heynig H, Mollenhauer D (eds) Süβwasserfora von Mitteleuropa, Vol. 19/1 Springer Spektrum, Berlin, pp 1–548

[21] Komárek J, Anagnostidis K (2005) Cyanoprokaryota. 2. Teil: Oscillatoriales. In: Büdel B, Gärtner G, Krienitz L, Schagerl M (eds) Süβwasserfora von Mitteleuropa, Vol. 19/2 Springer Spektrum, Berlin, pp 1–759.

[22] John DM, Whitton BA, Brook AJ (2003). The freshwater algal flora of the British isles: an identification guide to freshwater and terrestrial algae.

[23] Hofmann G, Werum M, Lange-Bertalot H (2013) Diatomeen im Süßwasser – Benthos von Mitteleuropa. Bestimmungsfora Kieselalgen für die ökologische Praxis. Über 700 der häufgsten Arten und ihre Ökologie. Koeltz Scientifc Books, Königstein

[24] Ettl H, Gärtner G (2014) Syllabus der Boden-, Luft- und Flechtenalgen. Springer Spektrum Berlin, Heidelberg

[25] Callahan BJ, McMurdie PJ, Rosen MJ, Han AW, Johnson AJA, Holmes SP (2016). DADA2: High-resolution sample inference from Illumina amplicon data. Nat Methods.

[26] Murali A, Bhargava A, Wright ES (2018). IDTAXA: a novel approach for accurate taxonomic classification of microbiome sequences. Microbiome.

[27] Wright ES (2015). DECIPHER: harnessing local sequence context to improve protein multiple sequence alignment. BMC Bioinformatics.

[28] Price MN, Dehal PS, Arkin AP (2010). FastTree 2–approximately maximum-likelihood trees for large alignments. PLoS ONE.

[29] McMurdie PJ, Holmes S (2013). phyloseq: an R package for reproducible interactive analysis and graphics of microbiome census data. PLoS ONE.

[30] Pavoine S, Dufour AB, Chessel D (2004). From dissimilarities among species to dissimilarities among communities: a double principal coordinate analysis. J Theor Biol.

[31] Anderson MJ (2001). A new method for non-parametric multivariate analysis of variance. Austral Ecol.

[32] Anderson MJ (2006). Distance-based tests for homogeneity of multivariate dispersions. Biometrics.

[33] Cao Y (2021) microbiomeMarker: microbiome biomarker analysis. R package version 0.0.1.9000. https://github.com/yiluheihei/microbiomeMarker. 10.5281/zenodo.3749415 Accessed 21 September 2023

[34] Love MI, Huber W, Anders S (2014). Moderated estimation of fold change and dispersion for RNA-seq data with DESeq2. Genome Biol.

[35] Benjamini Y, Hochberg Y (1995). Controlling the false discovery rate: a practical and powerful approach to multiple testing. J R Stat.

[36] Nguyen NH, Song Z, Bates ST, Branco S, Tedersoo L, Menke J, Schilling JS, Kennedy PG (2016). FUNGuild: an open annotation tool for parsing fungal community datasets by ecological guild. Fungal Ecol.

[37] Jehlička J, Edwards HGM, Osterrothová K, Novotná J, Nedbalová L, Kopecký J, Němec I, Oren A (2014). Potential and limits of Raman spectroscopy for carotenoid detection in microorganisms: implications for astrobiology. Philos Trans R Soc A.

[38] Ropret P, Tavzes Č, Retko K, Legan L, Špec T, Ocepek N (2012). Assessment of lichens’ metabolic and degradation products at Dornava Manor. Int J Herit Digi Era.

[39] Ricci C, Miliani C, Brunetti BG, Sgamellotti A (2006). Non-invasive identification of surface materials on marble artifacts with fiber optic mid-FTIR reflectance spectroscopy. Talanta.

[40] Frank-Kamemetskaya O, Rusakov A, Barinova E, Zelenskaya M, Vlasov D (2012) The formation of oxalate patina on the surface of carbonate rocks under the influence of microorganisms. Proc 10th Int Congr Appl Mineral 213–220. 10.1007/978-3-642-27682-8_27

[41] Russell JD, Jones D, Vaughan D, Fraser AR (1980). A preliminary study of fungal melanin by infrared spectroscopy. Geoderma.

[42] Schmaler-Ripcke J, Sugareva V, Gebhardt P, Winkler R, Kniemeyer O, Heinekamp T, Brakhage AA (2009). Production of pyomelanin, a second type of melanin, via the tyrosine degradation pathway in Aspergillus fumigatus. Appl Environ Microbiol.

[43] Capozzi V, Perna G, Gallone A, Biagi PF, Carmone P, Fratello A, Guida G, Zanna P, Cicero R (2005). Raman and optical spectroscopy of eumelanin films. J Mol Struct.

[44] Roldán ML, Centeno SA, Rizzo A (2014). An improved methodology for the characterization and identification of sepia in works of art by normal Raman and SERS, complemented by FTIR, Py-GC/MS, and XRF. J Raman Spectrosc.

[45] Strycker BD, Han Z, Bahari A, Pham T, Lin X, Shaw BD, Sokolov AV, Scully MO (2021) Raman characterization of fungal dhn and dopa melanin biosynthesis pathways. J Fungi 7(10):841. 10.3390/jof7100841 10.3390/jof7100841 PMC8540899 34682262

[46] Lombardozzi V, Castrignanò T, D’antonio M, Municchia AC, Caneva G (2012). An interactive database for an ecological analysis of stone biopitting. Int Biodeter Biodegr.

[47] Tomaselli L, Lamenti G, Bosco M, Tiano P (2000). Biodiversity of photosynthetic micro-organisms dwelling on stone monuments. Int Biodeter Biodegr.

[48] Caneva G, Ceschin S (2008) Ecology of biodeterioration. In: Caneva G, Nugari MP, Salvadori O (eds) Plant biology for cultural heritage: biodeterioration and conservation, Getty Conservation Institute, Los Angeles, pp 35–58.

[49] Macedo MF, Miller AZ, Dionísio A, Saiz-Jimenez C (2009). Biodiversity of cyanobacteria and green algae on monuments in the Mediterranean Basin: an overview. Microbiology.

[50] Pinna D, Edith J (2021). Microbial growth and its effects on inorganic heritage materials. Microorganisms in the deterioration and preservation of cultural heritage.

[51] Salvadori O, Municchia AC (2016). The role of fungi and lichens in the biodeterioration of stone monuments. Open Conf Proc J.

[52] Sohrabi M, Favero-Longo SE, Pérez-Ortega S, Ascaso C, Haghighat Z, Talebian MH, Fadaei H, de los Ríos A (2017) Lichen colonization and associated deterioration processes in Pasargadae, UNESCO world heritage site, Iran. Int Biodeter Biodegr 117: 171-182. 10.1016/j.ibiod.2016.12.012

[53] Esmaeillou M, Sohrabi M, Ofoghi H (2022). The study of the destructive roles of the endolithic lichen Verrucaria buschirensis s. lat., family Verrucariaceae on the World Heritage Site Persepolis. J Res Archaeo.

[54] Li Q, Zhang B, Yang X, Ge Q (2018). Deterioration-associated microbiome of stone monuments: structure, variation, and assembly. Appl Environ Microbiol.

[55] Hametner C, Stocker-Wörgötter E, Grube M (2014). New insights into diversity and selectivity of trentepohlialean lichen photobionts from the extratropics. Symbiosis.

[56] Kharkongor D, Ramanujam P (2015) Spatial and temporal variation of carotenoids in four species of Trentepohlia (Trentepohliales, Chlorophyta). J Bot Article ID201641. 10.1155/2015/201641

[57] Gaylarde P, Englert G, Ortega-Morales O, Gaylarde C (2006). Lichen-like colonies of pure Trentepohlia on limestone monuments. Int Biodeter Biodegr.

[58] Dias L, Rosado T, Candeias A, Mirao J, Caldeira AT (2020). A change in composition, a change in colour: the case of limestone sculptures from the Portuguese National Museum of Ancient Art. J Cult Her.

[59] Ortega-Morales BO, Gaylarde CC (2021). Bioconservation of historic stone buildings—an updated review. Appl Sci.

[60] Pinna D, Salvadori O (2000) Endolithic lichens and conservation: an underestimate question. In: Fassina V (ed) Proceedings of the 9^th^ International Congress on Deterioration and Conservation of Stone, Venice, pp 513–519. 10.1016/B978-044450517-0/50136-7

[61] Adamo P, Violante P (2000). Weathering of rocks and neogenesis of minerals associated with lichen activity. Appl Clay Sci.

[62] Bungartz F, Garvie LA, Nash TH (2004). Anatomy of the endolithic Sonoran Desert lichen Verrucaria rubrocincta Breuss: implications for biodeterioration and biomineralization. Lichenologist.

[63] Favero-Longo SE, Gazzano C, Girlanda M (2011). Physical and chemical deterioration of silicate and carbonate rocks by meristematic microcolonial fungi and endolithic lichens (Chaetothyriomycetidae). Geomicrobiol J.

